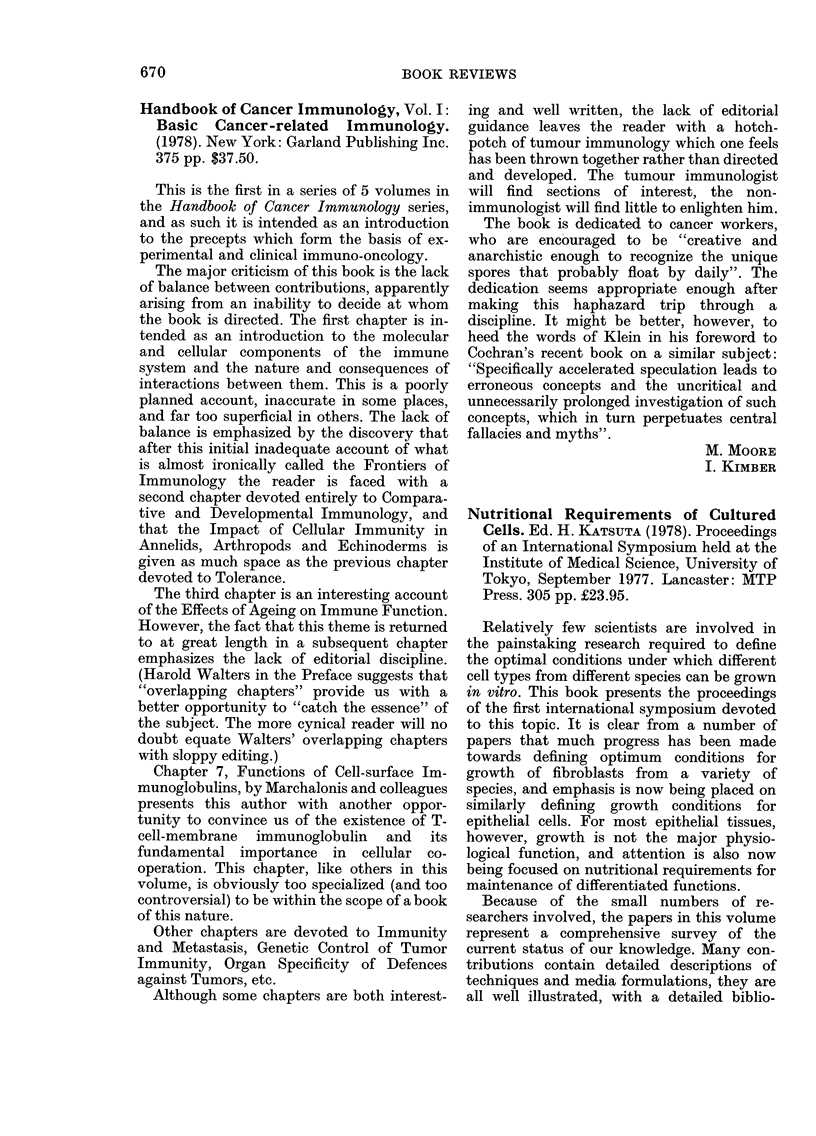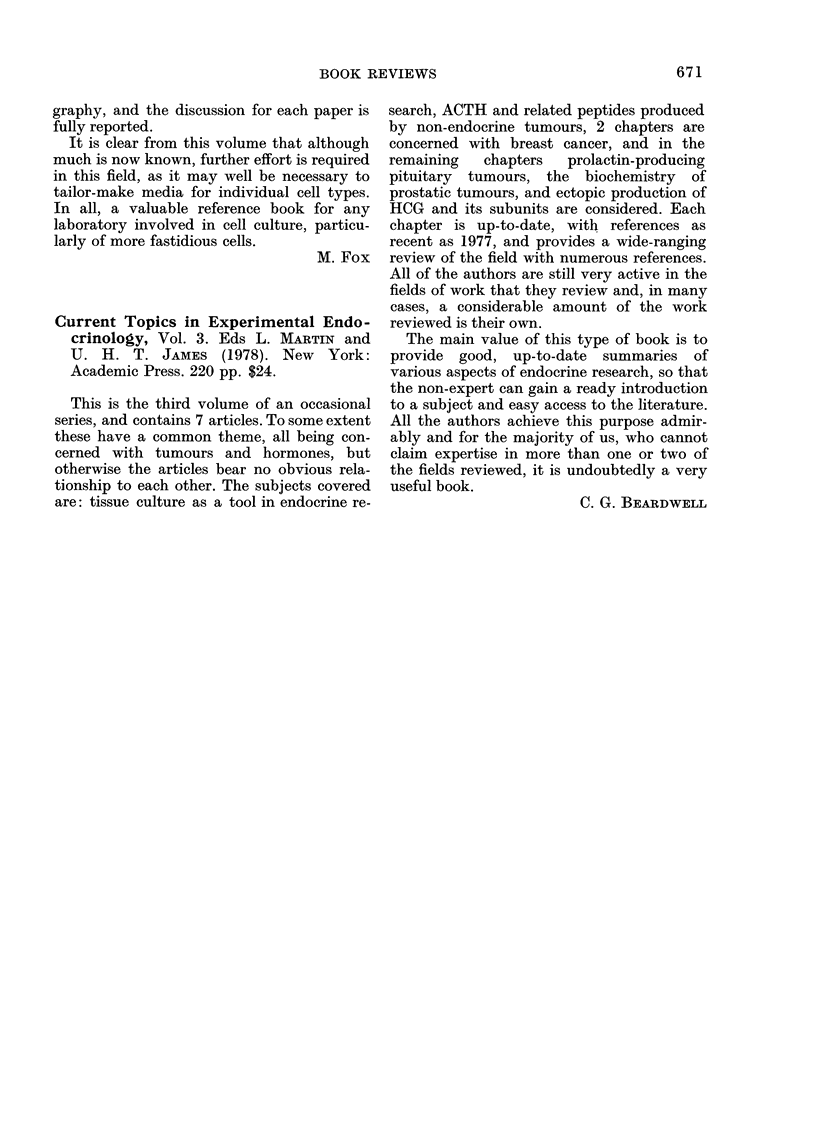# Nutritional Requirements of Cultured Cells

**Published:** 1979-10

**Authors:** M. Fox


**Nutritional Requirements of Cultured Cells.** Ed. H. Katsuta (1978). Proceedings of an International Symposium held at the Institute of Medical Science, University of Tokyo, September 1977. Lancaster: MTP Press. 305 pp. £23.95.

Relatively few scientists are involved in the painstaking research required to define the optimal conditions under which different cell types from different species can be grown *in vitro*. This book presents the proceedings of the first international symposium devoted to this topic. It is clear from a number of papers that much progress has been made towards defining optimum conditions for growth of fibroblasts from a variety of species, and emphasis is now being placed on similarly defining growth conditions for epithelial cells. For most epithelial tissues, however, growth is not the major physiological function, and attention is also now being focused on nutritional requirements for maintenance of differentiated functions.

Because of the small numbers of researchers involved, the papers in this volume represent a comprehensive survey of the current status of our knowledge. Many contributions contain detailed descriptions of techniques and media formulations, they are all well illustrated, with a detailed bibliography, and the discussion for each paper is fully reported.

It is clear from this volume that although much is now known, further effort is required in this field, as it may well be necessary to tailor-make media for individual cell types. In all, a valuable reference book for any laboratory involved in cell culture, particularly of more fastidious cells.

M. Fox